# Primary splenic histiocytic sarcoma associated with hemophagocytic lymphohistiocytosis: A case report and review of literature of next‐generation sequencing involving *FLT3* , *NOTCH2* , and *KMT2A* mutations

**DOI:** 10.1002/cnr2.1496

**Published:** 2021-07-22

**Authors:** Nelson Montalvo, Jorge Lara‐Endara, Ligia Redrobán, María Leiva, Christian Armijos, Leonardo Russo

**Affiliations:** ^1^ Facultad de Ciencias Médicas de la Salud y la Vida, Escuela de Medicina, Departamento de Docencia e Investigación Universidad Internacional del Ecuador, Av. Simón Bolívar y Jorge Fernández Quito Ecuador; ^2^ Servicio de Patología Hospital Metropolitano, Av. Mariana de Jesús s/n y Nicolás Arteta Quito Ecuador; ^3^ Servicio de Hematología Hospital Metropolitano, Av. Mariana de Jesús s/n y Nicolás Arteta Quito Ecuador; ^4^ Servicio de Radiología Hospital Metropolitano, Av. Mariana de Jesús s/n y Nicolás Arteta Quito Ecuador

**Keywords:** case report, *FLT3*, hemophagocytic lymphohistiocytosis, *KMT2A*, *NOTCH2*, primary splenic histiocytic sarcoma

## Abstract

**Background:**

Histiocytic sarcoma is a very rare monocyte/macrophage‐derived hematopoietic system tumor with a poor prognosis whose diagnosis is pathologically challenging due to its extreme rarity and histological overlap with various mimicking entities in which histiocytes also predominate.

**Case:**

We report the case of a 33‐year‐old male patient with hemophagocytic lymphohistiocytosis, purpuric syndrome, and significant splenomegaly. The patient underwent splenectomy; subsequent macroscopic examination revealed a spleen weighing 2065 grams with hyperemic red pulp and multiple infarcts at the periphery. The histological and immunohistochemical study established a diagnosis of primary splenic histiocytic sarcoma with frequent hemophagocytosis. Next‐generation sequencing demonstrated mutations in *FLT3, NOTCH2*, and *KMT2A,* microsatellite stability, and a tumor mutational burden of 2 mut/Mb. The patient's condition deteriorated clinically from the appearance of the first symptoms and he died 6 months later from multi‐organ failure.

**Conclusion:**

Primary splenic histiocytic sarcoma is one of the rarest tumors of the hematopoietic system. We report the first case with mutations in *FLT3, NOTCH2*, and *KMT2A,* and associated hemophagocytic lymphohistiocytosis.

## 1 INTRODUCTION

Histiocytic sarcoma (HS) is a very rare tumor of the hematopoietic system. The World Health Organization (WHO) classifies it among the hematopoietic and lymphoid system neoplasms derived from histiocytes or dendritic cells.^1^ It may be primary (of the skin, lymph nodes, digestive system, central nervous system, or disseminated) or secondary to another hematological neoplasm. There are no global epidemiological data concerning HS; however, the largest known case series, conducted by the U.S. National Cancer Institute, determined its overall incidence to be 0.17 per million people. Out of a total of 159 cases, only 5% were primary HS of the spleen and reticuloendothelial system.^2^ The analyzed literature describes the association of PSHS and hemophagocytosis without detailing the fulfillment of the diagnostic criteria to classify HLH secondary to PSHS. Because of its poor prognosis, early HS diagnosis is critical to improving patient survival, but such diagnosis is a clinical and pathological challenge because of the more than 100 recently described histiocytosis subtypes, varying between benign and malignant forms, that can only be differentiated through immunohistochemical, chromosomal, and molecular studies.^3^ We present the first case of HLH‐associated PSHS with an NGS study identifying *FLT3, NOTCH2*, and *KMT2A* gene mutations that suggest new therapeutic strategies.

### 1.1 Case presentation

**FIGURE 1:**
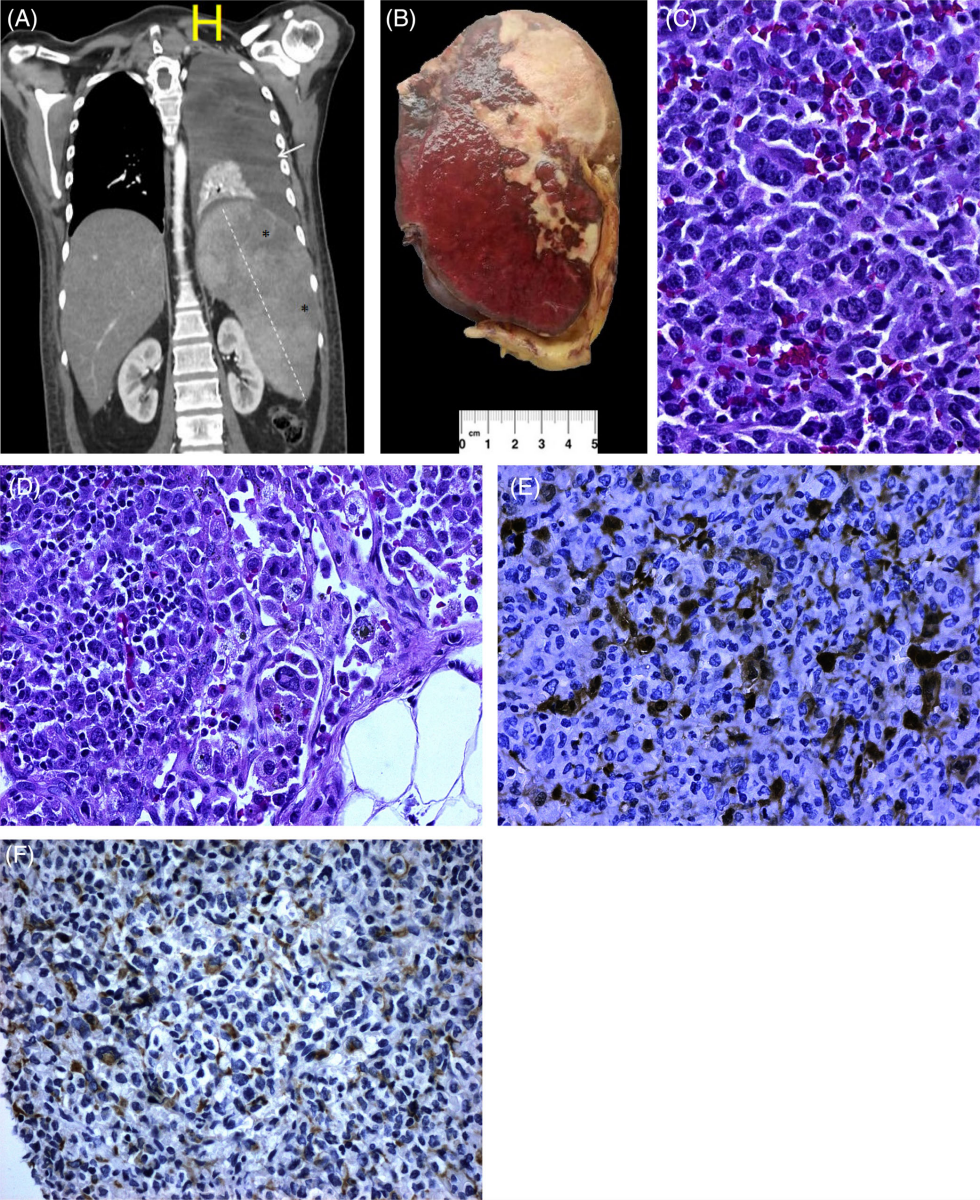
PSHS. (A) Simple and contrasted tomography showed a left pleural effusion, hepatomegaly, and supermassive splenomegaly with peripheral infarcts. (B) Multiple wedge‐shaped preferentially subcapsular ischemic infarctions. (C) Diffuse proliferation of medium to large neoplastic cells with pleomorphic nuclei, vesicular chromatin, prominent nucleolus and clear to eosinophilic cytoplasm, atypical mitotic figures, and apoptotic bodies [H/E 40×]. (D) Frequent hemophagocytosis produced by reactive and neoplastic histiocytes [H/E40×]. (E) and (F) The tumor cells showed positivity for S100 and CD68 [40×]

A 33‐year‐old Hispanic male patient without previous medical and family history of illness, came in for consultation regarding weight loss and fever. He was admitted to a hospital 4 months later with a diagnosis of HLH, multiple organ dysfunction, pneumonia, splenic infarction, and cytomegalovirus (CMV) infection. In the fifth month, he presented generalized pallor, purpura over the chest and lower limbs, edema in the legs, and hemiparesis of the left foot. Corticosteroids, intravenous immunoglobulin, and valganciclovir were administered. A simple and contrasted tomography showed a significant left pleural effusion reaching the pulmonary hilum, collapse of the ipsilateral lower lobe, hepatomegaly, and supermassive (2065 g) splenomegaly with peripheral infarcts (Figure 1(A)). No masses, retroperitoneal, mediastinal, or pulmonary hilar lymph node enlargements were found. Pleural and abdominal cytology tests were negative. Bone marrow flow cytometry showed no monoclonality, blasts, dysplasia, or myelofibrosis; neither acute myeloid leukemia nor Non‐Hodgkin lymphoma infiltration was found. The iliac crest bone marrow biopsy was hypocellular without hemophagocytosis. Splenectomy, distal pancreatectomy, and left adrenalectomy were performed in the sixth month. The electrophysiological study of the four extremities found distal symmetric axonal sensorimotor polyneuropathy. A control tomography revealed hepatomegaly (a liver volume of 5500 cc) without tumors, as well as preaortic, para‐aortic, and intercavo‐aortic adenopathies. The tumor was subjected to NGS (FoundationOne®Heme) to find possible therapeutic targets. The patient died of multiple organ failure 6 months after the onset of his condition.

### 1.2 Histopathological findings

We received the postsurgical samples of the spleen, distal pancreas, and left adrenal gland. Macroscopically, the spleen weighed 2065 g and measured 26 × 19 × 12 cm, with a grayish lobed capsule interspersed with firm whitish areas (Figure 1(B)). The section revealed no nodular lesions, but multiple wedge‐shaped, preferentially subcapsular ischemic lesions from 3 to 6 cm in diameter were observed, and 10 lymph nodes ranging from 0.2 to 0.6 cm in diameter were isolated. Microscopically, the spleen showed expansion of the red pulp cords and sinuses due to diffuse proliferation of medium to large neoplastic cells with pleomorphic nuclei, vesicular chromatin, prominent nucleolus and clear to eosinophilic cytoplasm, atypical mitotic figures, and apoptotic bodies (Figure 1(C)). In addition, we observed frequent hemophagocytosis produced by reactive and neoplastic histiocytes (Figure 1(D)). Furthermore, all examined lymph nodes showed lymphatic invasion and tumor involvement; acute pancreatitis was also found. Immunohistochemical stains showed tumor cell positivity for S100, CD68 (Figure 1(E) and (F)), CD45, CD4, lysozyme and CD56 and negativity for CD34, CD117, myeloperoxidase, CD8, CD20, CD1a, CD5, CD3, CD30, CD163, CD35, CD21, ALK, and pan‐melanoma panel. The rate of tumor cell proliferation (Ki‐67) was 70%. We ruled out myeloid differentiation due to CD34, CD117 and myeloperoxidase negativity. The established histopathological diagnosis was primary splenic histiocytic sarcoma (PSHS).

## 2 MATERIALS AND METHODS

We performed immunohistochemical staining on 4 μm formalin‐fixed and paraffin‐embedded (FFPE) tissue sections using the VENTANA Benchmark system (Roche, Tucson, AZ) according to standardized laboratory procedures. The following antibodies were used during the diagnostic study: CD68 (KP1), CD4, CD56, CD45, CD1a, CD163, CD8, CD3, CD5, CD20, CD30, CD35, CD21, S100, lysozyme, CD34, CD117, myeloperoxidase, ALK, pan‐melanoma panel, and Ki‐67.

### 2.1 Molecular profiling

A comprehensive genomic profiling test with the FoundationOne®Heme panel of genes was performed by Foundation Medicine, Inc. (Cambridge, MA) based on published methods. FoundationOne®Heme is designed to include genes known to be somatically altered in human hematologic malignancies and sarcomas that are valid targets for therapy, either approved or in clinical trials, and/or that are unambiguous drivers of oncogenesis based on current knowledge. This genetic profiling utilizes DNA sequencing to interrogate 406 genes as well as selected introns of 31 genes involved in RNA gene rearrangements and sequencing.

## 3 DISCUSSION

Part of the malignant histiocytosis group, HS is a hematolymphoid neoplasm classified as primary (in the skin, lymph nodes, digestive system, central nervous system, and disseminated) or secondary to a hematological neoplasm (follicular lymphoma, acute lymphocytic leukemia, or hairy cell leukemia), according to its location. It may also be subclassified according to the tumor's cellular origin (histiocytic, interdigitating dendritic, Langerhans or indeterminate cells, or of unspecified origin).^3^ Due to the possible common cellular origin and clinical similarities of histiocytoses, primary malignant histiocytoses may be properly diagnosed by first ruling out other tumors showing negativity for keratin, EMA, Melan‐A, HMB45, T and B lymphocyte markers, and follicular dendritic cell markers; and second, by expressing at least two of the following dendritic cell and/or histiocyte markers: CD68, CD163, CD4, and lysozyme. Cell subtype identification among malignant histiocytoses relies on stains such as S100, CD1a, and CD207.^3^ According to the WHO, the microscopic morphology of HS is characterized by diffuse proliferation of large and pleomorphic cells; neoplastic cells are round to oval with spindle‐shaped areas; and the nucleus is large, round to oval or irregular, and frequently peripheral, with mild to severe atypia and vesicular chromatin. The cytoplasm is abundant and eosinophilic with fine vacuoles, and hemophagocytosis may be present.^4^ Our patient's morphology, microscopy, and immunohistochemistry results meet the diagnostic criteria for HS.

HS is not necessarily associated with a B‐cell neoplasm, since these mutations have been observed in sporadic or primary cases of HS, despite the presence of clonal immunoglobulin rearrangements or translocations.^5^ C. Egan et al. performed a genomic study of 21 primary histiocytic sarcoma (PHS) cases that has helped to shed light on the differentiation of primary from secondary HS, showing that the RAS/MAPK pathway had the most frequent mutations in 19/21 cases; the most commonly altered genes were *NF1* and *MAP2K1*, with five cases each; and four of the five cases with *NF1* mutation involved the gastrointestinal tract. It should be noted that no primary splenic case was included.^5^

Our patient's NGS study reported *FLT3* and *NOTCH2* gene mutations, MSS, and a TMB of 2 mut/Mb. The MSS status and TMB data suggested that he probably would not benefit from immunotherapy and were concordant with those described by Goyal et al. through his prospective study of genomic biomarkers in 16 histiocytic neoplasms, which included a case of HS of the nasal cavity.^6^ It is important to note that the *BRAF* mutation was not found in our case, despite it has been described in other cases of HS.^5^

The fms‐related receptor tyrosine kinase‐3 (FLT3) is a type III tyrosine kinase that functions as a hematopoietic progenitor of cell proliferation, survival, and differentiation.^7^ Studies have shown that FLT3 activation is involved in leukemogenesis through serine/threonine kinase AKT phosphorylation or activation, which occurs in one‐third of acute myeloid leukemia cases and confers a worse prognosis.^7^ Presumably, FLT3 ligands can activate the RAS/MAPK pathway, and MAP kinase activation is needed for mitogenic signaling of FLT3.^8^ Despite the clear association between FLT3 and acute myeloid leukemia, we were unable to find any mention of a possible association between FLT3 and HS in our search of medical literature. Experiments in mice demonstrated that FLT3 is one of the regulators of dendritic cell progenitors in the bone marrow and peripheral dendritic cells; it is also essential in regulating homeostasis in splenic dendritic cell development.^9^ Despite the important role of *FLT3* in vivo, no overexpression of this gene was found in Langerhans cell histiocytosis (LCH).^10^ Bao et al. describe an unusual case of myeloid sarcoma (MS) of the vaginal wall having a morphological and immunohistochemical overlap with HS. NGS demonstrated *FLT3* mutation, but no medical literature was found that associates these two neoplasms. The findings in our case may suggest genomic links between MS and HS.^11^

Notch signaling pathway gain‐of‐function mutations are well‐known oncogenic drivers, especially for neoplasms such as T‐cell lymphoblastic leukemia, chronic lymphocytic leukemia, diffuse B‐cell lymphoma, mantle lymphoma, breast cancer, and lung cancer.^12^ *NOTCH2* plays a specific immunological role in splenic marginal zone cells and dendritic cells. Inadequate signaling of this pathway has been shown to play an oncogenic role in B‐cell and myeloid neoplasms.^13^ The specific NGS‐identified mutations of our patient have not been previously described in direct relation to PSHS, but Egan et al. described four cases of HS with Notch signaling pathway mutations including *NOTCH2*, demonstrating the possible relationship of this pathway with B‐cell lymphomas and, in particular, HS.^5^

In addition to the mentioned mutations, the variants of unknown significance (VUS) *ATM, LRKK2, MYO18A, SETBP1, NOD1, TSC1, NTRK1, CBL, NOTCH1*, and *KMT2* were described in our patient's tumor. These mutations had not been described at the time of the report (March 2020) and in light of their genomic context, their significance is not clear. Interestingly, Egan et al. describe mutations in cell cycle regulators, which include the ATM kinase. They also mention epigenetic modifier gene mutations and/or transcription factors in eight HS (*KMT2D*) cases.^5^ Epigenetic dysregulation in cancer manifests as global DNA hypomethylation, causing genomic instability and tumor suppressor gene and microRNA silencing through hypermethylation.^14^ The VUS *KMT2A/MLL1* was found in our patient. Mixed leukemia lineage (MLL) is a family of proteins whose function is histone‐H3K4 methylation to regulate active gene transcription.^15^ It is known that the *KMT2A* gene is necessary to generate an adequate number of hematopoietic progenitors.^16^ Mutation of this gene is found in 5–10% of adult acute myeloid and lymphocytic leukemias; such cases are characterized by poor prognosis and refractory to treatment.^14^ Bao et al. highlight the importance of *KMT2A* mutations and rearrangements found in MS with HS‐like morphology, leading us to consider the relationship that these two neoplasms may have in addition to their common oncogenic pathway. We have so far found no literature describing the possibility of transdifferentiation from HS to MS.^11^ We ruled out myeloid sarcoma among other entities due to our histological and immunohistochemical findings. Other authors have reported molecular similarities with variants like those of our patient *(NTRK1* in LCH), pointing to the possible importance of these mutations in histiocytosis and other hematological neoplasms.^17^

Our case is exceptional in that it is, to our knowledge, only the second reported case of PSHS with no macroscopic splenic nodules^18^ and constitutes an unusual presentation of HLH associated with a primary splenic tumor, which has been scantly described in the literature.^19^, ^20^, ^21^ Horny et al. described the association between HS and HLH in 1988, but the difficulty of differentiating the specific cause of HLH persists, because it can result from primary causes (hereditary Mendelian diseases) or secondary causes (infections, hematological neoplasms, solid tumors, and autoimmune diseases).^4^, ^20^

Despite the paucity of reported PSHS cases, neoplastic cells and reactive histiocytes have been shown to present phagocytosis in one or all hematological cell lines, including proteins such as immunoglobulins and albumin,^22^ and PSHS may thus be a secondary cause of HLH.^19^ Despite the possibility for neoplastic HS cells to produce hemophagocytosis, there is no information regarding the cause nor the consequence for reactive histiocytes to lose their physiological activity and start acting like neoplastic cells; we hypothesize that PSHS can be the responsible for the aberrant activity of histiocytic cells, which can range from normal to reactive, and finally neoplastic.^21^, ^22^ Furthermore, fulfillment of the Histiocyte Society HLH‐2004 diagnostic criteria^23^ has not been described in PSHS, possibly due to the extreme rarity of this neoplasm.

Our patient met the criteria for HLH (fever, splenomegaly, and platelets: 10 × 10^9^/L; fibrinogen: 49.3 mg/dL; triglycerides: 2271 mg/dL; ferritin: 5196 mg/dL; and splenic hemophagocytosis) secondary to PSHS during his first hospitalization. During his second hospital stay, he presented positive CMV IgM serology, indicating an opportunistic CMV infection. Epstein–Barr virus serology was negative. The patient's distal axonal sensorimotor polyneuropathy is likely to have been of paraneoplastic etiology, due to its presentation from the first hospitalization and prior to any treatment.

## 4 CONCLUSION

PSHS is a rare tumor of the hematopoietic system originating from dendritic cells. Its clinical and histopathological presentation is similar to other neoplasms with the same cell lineage, thus necessitating immunohistochemical and molecular techniques for proper diagnosis. We present the first report of PSHS with NGS‐identified mutations in *FLT3, NOTCH2*, and *KMT2A*. We suggest that these mutations may be key in the genetic origin of HS and in particular PSHS. We recognize the importance of epigenetic modifiers, such as KMT2A, as one of the possible oncogenic pathways for these neoplasms. A PSHS diagnosis should be considered as a possibility in patients presenting with HLH. Molecular studies have become an essential diagnostic tool that differentiates similar pathologies and identifies common molecular pathways to establish targeted therapies. Neither our patient's mutations nor the accompanying VUS have been described to date, and further NGS studies are therefore needed to demonstrate their genomic and clinical significance in the future.

**Abbreviations**
HS: Histiocytic sarcoma
HLH: Hemophagocytic lymphohistiocytosis
PSHS: Primary splenic histiocytic sarcoma
NGS: Next‐generation sequencing
MSS: Microsatellite stability
TMB: Tumor mutational burden
WHO: World Health Organization
CMV: Cytomegalovirus
muts/Mb: Mutations per megabase
MS: Myeloid sarcoma
LCH: Langerhans cell histiocytosis
VUS: Variants of unknown significance
PHS: Primary histiocytic sarcoma
MLL: Mixed leukemia lineage

## CONFLICT OF INTEREST

The authors have stated explicitly that there are no conflicts of interest in connection with this article.

## AUTHOR CONTRIBUTIONS

**Nelson Montalvo:** Conceptualization; data curation; formal analysis; funding acquisition; investigation; methodology; project administration; resources; supervision; validation; visualization; writing‐original draft; writing‐review & editing. **Jorge Lara‐Endara:** Conceptualization; data curation; formal analysis; investigation; methodology; writing‐original draft; writing‐review & editing. **Ligia Redrobán:** Conceptualization; formal analysis; investigation; methodology; project administration; resources; supervision; validation; visualization; writing‐review & editing. **María Leiva:** Conceptualization; formal analysis; investigation; resources; supervision; visualization; writing‐review & editing. **Christian Armijos:** Conceptualization; data curation; resources; writing‐original draft. **Leonardo Russo:** Conceptualization; investigation; writing‐original draft.

## ETHICAL STATEMENT

The article complied with the local ethics committee (Ethics Committee, Hospital Metropolitano, Quito, Ecuador) and adhered to the principles outlined in the Declaration of Helsinki. Written informed consent for publication of their clinical details and/or clinical images were obtained from the patient's next of kin.

## Data Availability

The data that support the findings of this study are available on request from the corresponding author. The data are not publicly available due to privacy or ethical restrictions.
